# Agile Six Sigma in Healthcare: Case Study at Santobono Pediatric Hospital

**DOI:** 10.3390/ijerph17031052

**Published:** 2020-02-07

**Authors:** Giovanni Improta, Guido Guizzi, Carlo Ricciardi, Vincenzo Giordano, Alfonso Maria Ponsiglione, Giuseppe Converso, Maria Triassi

**Affiliations:** 1 Department of Public Health, University Hospital of Naples Federico II, 80131 Naples, Italy; triassi@unina.it; 2 Materials and Production Engineering, Department of Chemical, University of Naples “Federico II”, 80125 Naples, Italy; g.guizzi@unina.it (G.G.); giuseppe.converso@unina.it (G.C.); 3 Department of Advanced Biomedical Sciences, University of Naples “Federico II”, 80131 Naples, Italy; carloricciardi.93@gmail.com; 4 AORN “Santobono-Pausillipon”, 80132 Naples, Italy; giordanovincenzo1961@gmail.com; 5 Department of Electrical Engineering and Information Technology (DIETI), University of Naples “Federico II”, 80125 Naples, Italy; alfonsom.ponsiglione@gmail.com

**Keywords:** agile, DMAIC, six sigma, modelling and simulation

## Abstract

Healthcare is one of the most complex systems to manage. In recent years, the control of processes and the modelling of public administrations have been considered some of the main areas of interest in management. In particular, one of the most problematic issues is the management of waiting lists and the consequent absenteeism of patients. Patient no-shows imply a loss of time and resources, and in this paper, the strategy of overbooking is analysed as a solution. Here, a real waiting list process is simulated with discrete event simulation (DES) software, and the activities performed by hospital staff are reproduced. The methodology employed combines agile manufacturing and Six Sigma, focusing on a paediatric public hospital pavilion. Different scenarios show that the overbooking strategy is effective in ensuring fairness of access to services. Indeed, all patients respect the times dictated by the waiting list, without “favouritism”, which is guaranteed by the logic of replacement. In a comparison between a real sample of bookings and a simulated sample designed to improve no-shows, no statistically significant difference is found. This model will allow health managers to provide patients with faster service and to better manage their resources.

## 1. Introduction

An increasing number of works deal with the problem of health management [1,2], which is a central issue in many political and social debates about the consumption of healthcare resources [3,4]. In this context, tools and methodologies, such as agile manufacturing, lean-agile manufacturing, and agile supply chains, promote a higher level of readiness for the continuous evolution of an extremely variable and unpredictable market [5,6]. For this reason, they are defined as “*market sensitive*” [7].

The literature offers numerous definitions of agile:

- Van Hoek et al. [8] defined agile as “*everything related to customer response and market turbulence that requires specific skills achieved using Lean Thinking*”.
- Robarts [9] defined it as “*the company ability to grow in a competitive and changeable market, to respond quickly to rapid changes in the markets driven by the improvement of products and services based on customer needs*”.
- Furthermore, Lee defined it as “*a set of strategies that solves the problem of uncertainty and the variability of demand by increasing the flexibility of the system*” [10].

Some common aspects emerge from these definitions: sensitivity to the environment, the exploitation of customer impact, and variability leaning through flexible strategies.

Carvalho et al. [11] analysed the differences and similarities between lean, agile, resilient, and green (LARG) methodologies. Lean is focused on flexibility and on the reduction of costs through the continuous elimination of wastes. It brings high returns and minimizes the inventory throughout the chain. Moreover, it shortens the lead time without increasing costs. Conversely, agile systems are basically focused on the rapidity of changes to guarantee higher flexibility.

In the healthcare system, the concepts of flexibility, modularity, and readiness make it possible to strengthen the response system for patient clients. Health managers need new service management strategies that are effectively responsive to changes and the negative effects of variability in patient care demand. Improvement consists of increasing the level of governance and control of a process and in decreasing errors through by analysing the causes that generate them.

In healthcare and in other sectors, lean and agile principles can be combined with the Six Sigma methodology to improve the performance, quality, and response of a process through data analysis. The premise of a Six Sigma project is the definition of a measurable quantitative objective. Therefore, it is fundamental to determine a relationship between the quantity to be improved (e.g., number of defects, the length of waiting lists, the use of a resource), generally called Y, and the input variables of the process (e.g., unique centre of booking (UCB) programming, status of equipment and resources), generally called X [12]. The correct definition of the answer (Y) and the discovery of the fundamental causes (X) require a complete knowledge of the process to be improved, a great analytical attitude, and the correct application of a certain number of qualitative and quantitative investigation tools, such as process maps, cause and effect matrices, failure mode and effects analysis (FMEA), and the define–measure–analyse–improve–control (DMAIC) cycle. DMAIC is one of the most effective tools for problem solving and constitutes the roadmap for project development.

Regarding the combination of lean and Six Sigma, many efforts have been made by researchers in the management of hospital processes through the application of lean Six Sigma and the DMAIC methodology. Southard et al. [13] studied the introduction of radio frequency identification (RFID) technology for surgical procedures. Similarly, also concerning surgical procedures, Improta et al. [14,15,16,17] analysed the implementation of fast track surgery for patients undergoing prosthetic hip replacement surgery through the DMAIC cycle. In contrast to the previous authors, Vijay did not focus on surgical procedures but applied the same methodology to improve the hospital discharge process [18]. Moreover, recently, Six Sigma has been used for software development, with the aim of eliminating waste-inducing defects [19], as reported in the works of Hamid et al. [20] and Asghar et al. [21].

Notably, regarding the combination of agile and Six Sigma, the agile approach is based on the daily collaboration of team members and focuses on observations and short-term improvements, neglecting decision-making based on measurable data. The integration of Six Sigma will provide agile teams with a structured methodology as well as tools for empirical problem solving, as reported by Safaie [22]. According to Alipour et al. [23], agile complements both lean thinking and Six Sigma methodologies by adding to them responsiveness and adaptability. Lean Six Sigma emphasizes project work on the identified variation from the proposed standard without focusing on the voice of customers. The combined implementation with agile improves this weakness. Moreover, Felhmann and Santillo [24] showed that merging a standard measurement method such as Common Software Measurement International Consortium (COSMIC) function points with Six Sigma within the agile development framework produces numerous benefits and advantages. Finally, Morris [25] examined the relationships of some methodologies, such as agile, lean production, and Six Sigma.

### 1.1. Patient No-Shows

Patient no-shows have been recognized as a critical issue within modern outpatient healthcare systems. Many studies have revealed a no-show rate higher than 10%. Deyo and Inui noted that the no-show rate generally ranged from 15% to 30% for both adult and paediatric patients, while Rust and Oliver reported that the rate of absentee patients for public paediatric facilities also reached 80% [26,27]. Common impacts include reduced clinical efficiency and low productivity, wasted medical resources, increased healthcare costs, and limited access to patient care.

As reported by Guzek et al. [28], academic medical centres (AMCs) take care of 26% of all medical hospitalizations but account for only 5% of hospitals. The yearly reimbursement loss because of missed appointments in a paediatric neurology clinic with a 26% no-show rate was $257,724.57 during the July 2013–June 2014 academic year (numbers based on estimated reimbursements). Billing losses amounted to $788,733.58, representing lost opportunities to improve patient access to care and to expand other parts of an AMC’s mission, including education.

Patient no-shows create unexpected downtime for medical personnel that is unrecoverable. Many efforts have been made by hospitals to reduce the rate of absenteeism: reminder letters, telephone calls, text messages, and even disincentives such as advance payment for the service, but the problem continues to be substantial [29]. Therefore, the development of proactive solutions, which identify patients’ failure to present and reduce the negative impact, is a fundamental task for improving the delivery of health services [30,31,32]. Various scheduling methods have attempted to reduce waiting lists and the negative impact of absentee patients. As a result, some researchers have proposed overbooking as an effective approach to handle the criticalities of absenteeism [33].

For example, overbooking was carried out to mitigate absenteeism in a health centre with 18 outpatient clinics in Denver (USA) through a lean Six Sigma approach [34,35,36]. Through lean, gaps and waste were identified in the process, while with Six Sigma, the desired target status, the causes of variability, and the intervention policies to reduce the gaps were described. Finally, overbooking was adopted to reduce patient no-shows.

Similarly, other examples regarding the implementation of overbooking can be found in the literature [37,38], such as the work of Liu and Ziya [39], where two different scenarios were compared: in the first scenario, they assumed that the daily service capacity is fixed and that the clinic does not have the option of overbooking patients; thus, the only decision variable is the arrival rate of patients, and the objective is to maximize the average number of patients served per day. In the second scenario, they assumed that the clinic’s service capacity is flexible (the clinic can opt for overbooking), and it represents a decision variable together with the arrival rate. After the characterization of both scenarios, the optimal decisions and their influence on patients’ show-up probabilities were identified, and the overbooking decision proved to play an important role in the management of the appointment scheduling policy.

Zacharias and Pinedo [40] presented an overbooking model for scheduling the arrivals of patients who have different no-show probabilities and different weights, exploring the trade-offs between the benefits of efficient physician utilization and the costs of patient waiting time. They demonstrated that the no-show rate and patient heterogeneity have a significant impact on the optimal schedule.

Samorani and La Ganga [41] dealt with the problem of scheduling outpatient appointments given individual day-dependent no-show predictions, and they found that when overbooking occurs, while it is desirable to maximize the number of patients seen, it is desirable to limit patient waiting time and clinic overtime. To solve this trade-off, they developed a dynamic scheduling procedure that considers no-show predictions, tested it via simulation, and validated it using the dataset of a large mental health centre. They showed that the open access approach would lead to a profit increase of up to 18% and that for low no-show rates, the quality of prediction should be shifted towards high sensitivity, while for high show rates, it should be shifted towards high specificity.

### 1.2. Objective of the Study

Within this framework, this study aims to improve patients’ access to clinical assistance in a paediatric hospital in southern Italy. To that end, we analysed all the activities carried out in the UCB of the hospital and investigated solutions to improve the scheduling policy of the UCB. An agile Six Sigma approach was implemented following the DMAIC cycle to identify process gaps and to measure performance improvements. A model was developed to simulate, examine, and evaluate different strategic choices adopted to manage and reduce patient no-shows.

## 2. Materials and Methods

This project was conducted at the Presidium Hospital of Naples AORN Santobono Pausilipon, which is a representative healthcare structure that is always active in facilitating access to services and reducing waiting times, in accordance with regional and national strategic directions. The strategic mission of the hospital is to satisfy the assistance needs of all paediatric citizens, guaranteeing services of effective, efficient, and timely prevention, treatment, and rehabilitation, provided with respect for the person and in conditions of absolute security. At the hospital, there are approximately 15,000 admissions per year (averaging the last 10 years), and it performs more than 100,000 outpatient services; additionally, it has approximately 400 beds. The hospital’s activities are carried out at two facilities:

- Santobono Hospital, divided into four pavilions: “Santobono”, “Torre”, “Volano”, and “Ravaschieri”, located in Naples;
- Pausilipon Hospital: located in Naples.

Booking services are managed by the UCB of the hospital, which is open Monday to Friday 8:00–17:30 and Saturday 8:00–12:00 and has three front office workers during the morning shift (8:00–14:00) and one employee in the afternoon shift (14:00–17:30). After booking, admitted patients are directed to the “Volano” pavilion, where outpatient services and activities are performed.

The issues raised by the general management of the hospital can be summarized in the following points:

1. Management of absentee patients and changes in and cancellations of services; and
2. Waiting lists.

To achieve the project objective, i.e., identifying and mitigating inefficiencies in patients’ access to services, the DMAIC cycle was used. We started from an analysis of inefficiencies in the access routes to the outpatient services performed by the structure. Through the different DMAIC phases, it was possible not only to identify critical aspects and the causes of the process inefficiencies but also to plan and implement improvement actions, with the aim of eliminating or mitigating the effects due to entropy resulting from transfers, cancellations, and no-show patients, and to make the process fair from the perspective of access to services.

Data were collected in the January 2016–December 2016 period and include the following information:

- Description of the visit booked (operative unit, department, type of service);
- Number of visits booked;
- Date and time when the visit is booked;
- How the visit is booked (over the phone/in person/other);
- ID booking;
- ID patient;
- ID visit;
- ID acceptance;
- Date and time when the visit is scheduled;
- Date and time when the patient is admitted;
- Time when the visit is assigned to the patient;
- Time when the patient is assigned for the visit; and
- Type of patient (chronic/first visit/other).

During the measure and analysis phases, the percentages of absenteeism of patients (i.e., patients who were not present at the time of the scheduled visit) were calculated for each unit, in addition to the mean, min, max, and standard deviation of the waiting time. Furthermore, other parameters (chaos, inefficiencies, potential, improvement, and waiting list reduction) to measure the process performances were calculated as follows.

Chaos is considered a synonym of entropy; thus, it is calculated by considering how many procedures are cancelled, shifted to another day, or not performed due to absenteeism Equation (1):

Chaos = (#No-Show + #shift + #cancel)/(#booking).
(1)

Equation (2) represent inefficiency, which is calculated as the difference between capability (number of visits that could effectively be performed) and the number of bookings, normalized by capability:

Inefficiency = (Capability − #booking)/Capability.
(2)

If the daily availability of booking is saturated, the number of visits that could be performed is calculated based on Equation (3):

Potential = Useful outpatient days * Availability.
(3)

The difference between the potentiality of the system and what it actually achieves, compared to its availability, provides the theoretical improvement in terms of the number of days useful for carrying out visits Equation (4):

Improvement = (Potential − Realized)/Availability.
(4)

To have the data in terms of a waiting list, improvement must be multiplied by 7/3 since visits are available for only 3 days of the week Equation (5):

Waiting list reduction = Improvement * (7/3).
(5)

This reduction can be achieved with the logic of scientific overbooking (as reported in the Results section).

After the analysis of the service-providing process, the description of the mechanism of operation, its characteristics, the logic in force, and the critical issues, the delineation of a conceptual model of access routes and performance executions is successfully completed. At this point, through a formal model, we could reproduce what we have mentally defined, thus simulating reality in analysis. Through the discrete event simulation (DES) approach, this conceptual model was materialized in a formal model that made it possible to represent the reality of the organization under study through a simulation model [42].

DES is a process of codifying the behaviour of a complex system as an ordered sequence of well-defined events. Known applications include stress testing, assessing possible financial investments, and modelling procedures and processes in the manufacturing and healthcare context. In healthcare, Baril et al. [43] reduced patient delays in an oncology clinic by 79% after 19 weeks, while Glover et al. [44] highlighted the benefits of using DES in a case study of abdominal aortic aneurism. Zhang [45] conducted a systematic review to explore the current advances and extent of DES, identifying four major areas: health and care systems operation, disease progression modelling, screening modelling, and health behaviour modelling.

Finally, based on the collected data, we virtually simulate the model by formulating ad hoc strategies to respond to the chaos resulting from absentee patients, shifts, and cancellations.

## 3. Results and Discussion

### 3.1. Define

First, the team dealing with the project was defined as follows: a medical manager was the project leader, a biomedical engineer was the project champion, and the team was composed of three engineers. While the leader and the champion dealt with conceptual issues, the team collected and analysed data; each member had to make a substantial contribution to the work.

**Figure 1:**
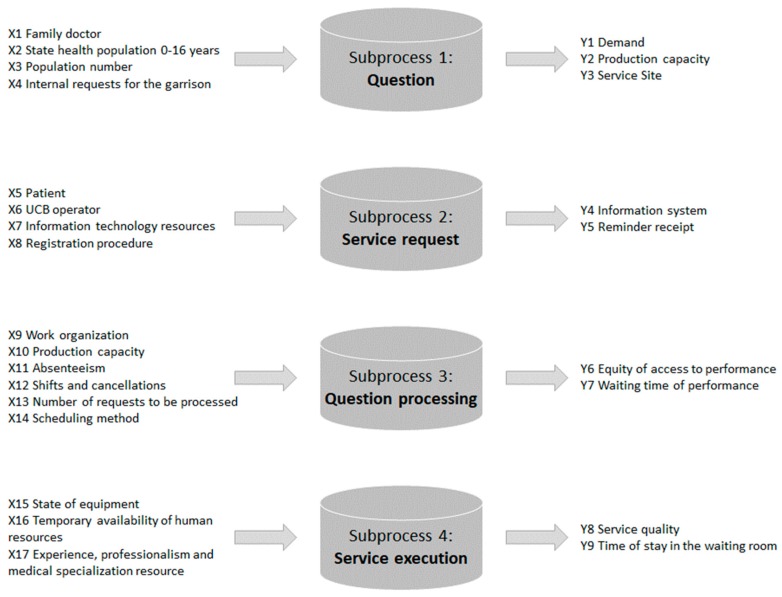
Map of the process.

To determine the relations between the quantities to be improved (Y) and the variables in input (X) able to influence their value, a detailed mapping of the process was carried out (Figure 1).

A project charter was developed with all the necessary details of the project:

- **Project title:** Agile and Six Sigma to reduce patient absenteeism.
- **Question:** Excessive absenteeism in the Presidium Hospital.
- **Critical to quality (CTQ):** X9 Work organization, X14 Scheduling method, X12 Shifts and cancellations, X15 State of equipment, X11 Absenteeism, X10 Production capacity.
- **Objective:** To realize corrective measures to reduce/increase the CTQ elements.
- **Team members:**
- **Timeline:**
  Define: August 2016
  Measure: September 2016
  Analyse: October 2016
  Improve: November 2016
  Control: November–February 2017
- **Within scope:** Absenteeism of patients within the hospital context.
- **Out of scope:** Other management problems regarding medical visits.

### 3.2. Measure

After the presentation of the variables in the define phase, in this phase, a decision is made concerning their inclusion and exclusion. According to the management of the hospital, the variables to be improved were selected, and a weight for each of them was provided: Y3 service site, Y4 information system, Y6 equity of access to performance, Y7 waiting time of performance, and Y9 time of stay in the waiting room were chosen by the director of the hospital as the target variables to be improved. A priority matrix makes it possible to select those critical variables that affect the output to be improved. For each of the input variables (X), a score was calculated considering their contributions to the output variables (Y); then, a cumulative score was computed.

**Figure 2:**
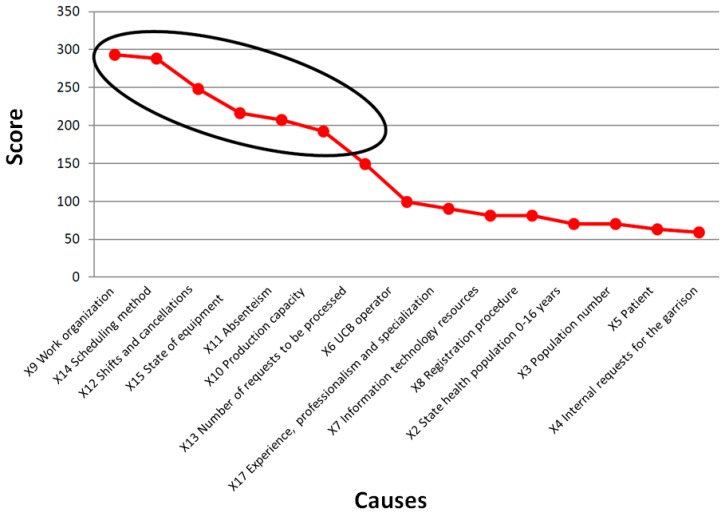
Calculation of the cumulative score. The circle in the graph indicates the variables that represent 80% of the critical issues.

By calculating the cumulative score based on the priority matrix, it was possible to identify which input variables contributed to approximately 80% of the critical issues (Figure 2): X9 Work organization, X14 Scheduling method, X12 Shifts and cancellations, X15 State of equipment, X11 Absenteeism, and X10 Production capacity. These variables represent the CTQ elements in the DMAIC approach.

### 3.3. Analyse

A Pareto analysis highlighted those performances that have the greatest impact in numerical terms.

The total number of visits, belonging to 198 different services, amounts to 66,649. These visits were booked and scheduled in 2016.

Eighty percent of the above sample (66,649) is represented by 21% (43 different performances) of the visits.

Regarding the services obtained, the following are calculated:

- **Average waiting time:** The difference between the agenda date (or date of referral) and the booking date, expressed in days;
- **Standard deviation:** The dispersion of waiting time with respect to its average value, expressed in days;
- **Max:** The maximum waiting time recorded for the performance in question, expressed in days;
- **Min:** The minimum waiting time recorded for the performance in question, expressed in days; and
- **Total:** The number of services booked.

Please note that the value of waiting time is approximated for those services rather than for general visits or first visits because the system does not record the date on which the visit should be scheduled as a direct consequence of a medical referral.

In our study we examined the following eight areas:

- Otolaryngology,
- General neurology,
- Emergency surgery,
- General orthopaedics,
- Infant neuropsychiatry,
- Dermatology,
- Medical day hospital,
- Cardiology.

The Absenteeism rate and Waiting times for the services provided by each area were measured with the following results: the Absenteeism rate ranged from 13% of the Emergency surgery to 38% of the Infant neuropsychiatry; the maximum Waiting time ranged from 182 days of the orthopaedic to 421 days of the Infant neuropsychiatry, while the mean Waiting time ranged from 16 days of the Emergency surgery to 208 days of the Infant neuropsychiatry.

The Chaos (computed as the percentage of no-show, shifts, and cancellations) and the Inefficiency of the process (computed as the percentage of services not compliant with the times established by law) were also computed for all the areas in order to understand which one of them affected the hospital. The Chaos and Inefficiency of the process are reported below for each examined area:

- Otolaryngology: 31% and 20%.
- General neurology: 25% and 30%.
- Emergency surgery: 47% and 3.3%.
- General orthopaedics: 34% and 24%.
- Infant neuropsychiatry: 56% and 85%.
- Dermatology: 53% and 42%.
- Medical day hospital: 49% and 54%.
- Cardiology: 33% and 19%.

After the analysis, due to the above-mentioned measurements, the Infant neuropsychiatry has been identified as a representative case to be improved, since it shows the highest value of both Chaos and Inefficiency of the process, respectively 56% and 85% (Table 1). Therefore, our simulation model has been tested on this representative area.

### 3.4. Simulation

From the analysis of the ambulatory service process of the hospital presidium, it was possible to outline the conceptual model of the department through the DES approach.

The modelling and simulation phases are reproduced and implemented on a computer to test different strategic solutions that make the model evolve towards successful solutions. Controlling the independent variables, it is possible to identify among different evolutions those to which the best business scenario corresponds [42].

The critical parameters of the model, i.e., those that must be monitored, are as follows:

- Waiting list of the service;
- Minimum waiting time;
- Maximum waiting time; and
- Waiting steady time: the time needed to work through the entire queue.

Based on these parameters, it is also possible to monitor the fairness of access to services. The following are the different design phases for conducting a simulation:

- **Capacity calendar creation:** This is defined as the sequence of days, in Julian format, in which the availability of the service to be simulated is open. Formally, the calendar is an n x 2 matrix, where n are the simulated days and for each day, the date and value of available services are shown—that is, the maximum number of visits expected on that particular day. From the real system, on average, the daily scheduled bookings are equal to 11, i.e., normally 10 plus an additional unit of overbooking.
- **Booking calendar creation:** This is defined as the sequence of the total bookings made by patients on different days of the week during a time window of amplitude necessary to work through the entire queue. To generate this calendar, first, the total daily bookings, recorded by the real system, were checked in the January–December 2016 time window and on different days of the week. This control was used to evaluate the homogeneity of bookings for the “day” factor and for the “month” factor.

For the data available, which belong to several groups (number of bookings on different days of the week and the year), it is necessary to understand whether they are “different” from each other or are part of the same “population” and whether their differences are due to normal statistical variability. “Different” means that the data are samples of a population described by a statistical distribution that is different for each of the groups.

**Figure 3:**
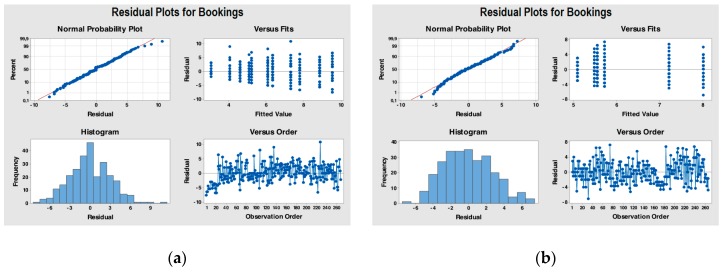
(**a**) Residual Plots for the variable “bookings” grouped according to the factor “month”; (**b**) Residual Plots for the variable “bookings” grouped according to the factor “day”. Visual inspection of the normal probability plot and the histogram seems to indicate that the residuals follow a normal pattern for both.

**Figure 4:**
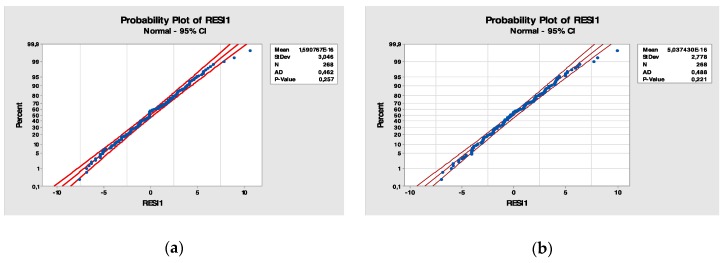
(**a**) Probability plot of the residuals for the variable “bookings” grouped according to the factor “month”; (**b**) Probability plot of the residuals for the variable “bookings” grouped according to the factor “day”.

Statistical analysis provides us with methods for interpreting these possible differences: the ANOVA method, based on comparing variances (Figure 3 and Figure 4).

One-way ANOVA for both factors produced the following results (Table 2 and Table 3).

Analyzing the ANOVA results, it is possible to conclude that since the *p*-value (0.000) associated with the F test is lower than the chosen significance level (0.05), the homogeneity hypothesis must be rejected. Therefore, it is possible to conclude that the bookings vary significantly (with significance level α = 0.05) depending on the month of recording.

Since the *p*-value (0.079) associated with the F test is higher than the chosen significance level (0.05), the hypothesis of homogeneity of the bookings with respect to the “day” variable can be accepted.

Following the ANOVA results and considering that only one sample is held, the non-parametric bootstrap method is applied to generate the bookings, which are of finite size and of which the distribution is not known.

### 3.5. Model Validation

In the next phase, it is necessary to check whether the model that has been created provides valid results for the system under study. In particular, the measurements of the real system must be well approximated by the measures generated by the simulation model.

The calibration process was followed by a model validation process or a “quality control” operation in which the capacity of the calibrated model to reproduce the real operation of the system is ascertained. In this phase, a comparison between real observations and simulated values was made using indicators of goodness of fit, with the aid of appropriate graphical representations.

In particular, the Kolmogorov–Smirnov (K-S) test was used. This test is a non-parametric test that allows one to compare a sample of data and a theoretical distribution or two samples of data to verify the statistical hypothesis that the population from which the data come are from the one in question or the hypothesis that both samples come from the same population, as in our case.

The comparison was made between the real sample and the simulated sample for the executed and absent visits variables. The real sample refers to the infant neuropsychiatry visit services in the August 2016–December 2016 time window, the window from which the data were extracted.

Kolmogorov–Smirnov Two-Sample Test.

- K-S test statistic: 0.160.
- K-S critical value (approximately): 0.254.
- Alpha level: 0.05.

The test statistic is less than the critical value; therefore, there is not sufficient evidence to conclude that the underlying distributions are different.

### 3.6. Improve

The following table (Table 4) shows the following for each level of overbooking:

- **#Under:** The number of visits below the expected availability;
- **#Over:** The number of visits exceeding the expected availability;
- **Average under:** This considers those visits below the expected availability, evaluating the difference between the simulated value and the real value. Average under is the average value among the registered gaps;
- **Average over:** This considers those visits above the expected availability, evaluating the difference between the simulated value and the real value. Average over is the average value among registered gaps;
- **Diff.:** The absolute value of the difference between the average under and the average over;
- **Equilibrium:** The average value among the gaps between the average under and the average over. If one imagines that the overbooking level is a faucet, positive values of the equilibrium indicate an overflow and, therefore, overbooking; in contrast, negative values indicate a clogged or malfunctioning faucet;
- **Steady time:** The number of days required to work through the entire queue.

A total of 187 visits over 112 useful days must be covered. Currently, the availability includes 10 visits a day; if the specialist in question works 8 h per day, the visits performed every single hour are those expressed in Equation (6).

Visits/Hours = Availability/(Working hours per day) = 10/8 = 1.25.
(6)

The actual time expressed in medical hours is described by Equation (7).

Hours/Doctor = (Visits to cover)/1.25 = 187/1.25 = 149.6.
(7)

The use of this project guarantees that all the visits are booked; therefore, overbookings are also carried out.

If the management of the hospital does not intend to adopt the “objective project” as a solution, the level of overbooking to be implemented must also consider other parameters. In fact, in the first analysis, the choice would fall on scenario 1, as it presents the lowest value of occurrences. However, this variable is not sufficient because it does not take into account the value of equilibrium and the possible overtime.

The balance leads to the exclusion of those scenarios in which the equilibrium is far from its ideal value (i.e., far from zero). Therefore, our attention is focused on scenarios 4–7 (Table 5), which have equilibrium values ranging from −1 to +1.

If one unit of overtime that covers two visits is considered tolerable, the overbookings become as shown in Table 6.

Scenario 4, which provides an overbooking level of 21 performances, is the best in terms of balance, occurrences, and visits covered by overtime.

### 3.7. Control

The control phase plays a key role because the process, if left to itself (without control), tends to degrade over time because it is influenced by external factors [46]. To be more responsive to the environment, the parameters that make it possible to define the level of overbooking must be constantly monitored and possibly modified, as a situation could be created in which the daily availability is exceeded, and vice versa. A continuous monitoring system makes it possible to understand in a preventive way when the parameters change significantly, directing the process towards a drift condition and then acting on them before non-conformities, represented by excessive overtime, occur. In this context, the dynamic overbooking presented in the literature is the most effective solution.

## 4. Conclusions

Adding agile to the Six Sigma approach has helped achieve a faster deployment of the model than would have been possible without the agile approach. The scheduling of visits and the timing of each task were dictated by the agile methodology, while the structure of the improvement process was followed based on the Six Sigma methodology. Indeed, both were characterized by deeply listening to the needs of customers (in this case, the hospital) and having an attitude oriented towards statistical thinking. It is important to highlight that the process underwent many analyses regarding variables to include, service to be considered, and model validation.

In this work, the aim of which is to identify and mitigate the inefficiencies in accessing services, we focus, from an agile perspective, on a preliminary analysis of the different constitutive stages of the services, carrying out a mapping of the different phases; moreover, we identify any redundancies and process losses. The progress process has established a discrepancy between what is foreseen by the procedures and what is actually implemented. Lean logic, which includes reservations by appointment, best deals with the problem of inflow and, therefore, safety at the hospital. However, it is not applicable in reality due to the external variability caused by absentee patients who distort the process.

National trends over the last three years, with high average no-show rates ranging from 17.1% to 19.3% [47], have shown that despite reminder phone calls, postcard reminders, direct cell phone calls, and administrative charges for patient no-shows aimed at addressing and altering no-show rates [48,49], the no-show phenomenon is still a challenging issue both nationally [50] and internationally [51]. Although using an overbooking strategy can improve revenue and clinical resource utilization, there is limited evidence documenting that the overbooking strategy is a positive contributor to lowering no-show rates because overbooking is beneficial only under certain conditions [39]. Indeed, overbooking can significantly increase the clinic service volume by increasing the availability for patients and the overall productivity of the clinic, leading to a reduction in costs and improved patient satisfaction. However, overbooking can prolong queues in clinics and increase patient dissatisfaction prior to the appointment at hand. However, the bottle neck was not represented by the low number of medical resources but by their unsaturation. Dimensioning the overbooking in the correct way, keeping into consideration the entire process (no-show, cancellations and movements), would allow hospital to obtain a substantial improvement.

Therefore, the use of such a simulator to choose whether or not to adopt overbooking and, above all, what level of overbooking is recommended could be helpful for the lean management of healthcare organizations. Indeed, the simulator could suggest the best strategy for the management of the booking calendar, reducing unbalanced waiting times between the booking and the scheduling of visits and granting equity to patients and a more timely and fair delivery of healthcare services.

The proposed simulation model for overbooking appointments proved to be an effective method of dealing with no-shows. The agile methodology was useful for fine tuning the parameters related to overbooking. The final reports of the hospital showed that the overbooking strategy, based on the total external disturbance caused by travel, cancellations, and no-shows, is effective in guaranteeing fair access to services. In this situation, in fact, all patients respect the times dictated by the waiting list, without “favouritism” guaranteed by the logic of replacement. Substantially, equity with regard to the order of patients who booked medical visits is guaranteed. In the presence of critical issues with waiting lists, the most useful intervention with an immediate impact consists of providing economic incentives to handle the criticality: overtime work.

Agile logic requires that the clinical staff organize some meetings to understand whether there is a period with a major influx of patients (such as an epidemic or a simpler “flu period”). This kind of situation will reduce absenteeism, and clinical staff should be prepared by having the correct amount of resources.

Regarding the results of the implementation of the model, the simulation of healthcare processes will benefit both the managerial side of hospitals and, of course, patients. The former will achieve a better management of critical situations such as absenteeism, while the latter will benefit from a shorter waiting time since more medical examinations will be available daily, reducing absenteeism. Of course, the simulation of processes will also affect the management of resources. There are also advantages that are related to the methodology we implemented: there is not the need to experiment the change in the real practice because it is possible to simulate it and quantify the economic consequences in advance; there is the possibility to experiment the improvement more than once and in different ways so that the process can be deeply understood; the time required for a real experiment is much greater than the one necessary for a simulation; ethics and law are not a problem when just making a simulation so the bureaucracy is not a limitation; finally, risky ideas can be implemented and tested without perturbing the real system.

### Limitation of the Study and Future Developments

Of course, there are some limitations of this study. The focus of this paper was mainly the creation of a simulation model to deal with patient no-shows. Therefore, classical statistical pre–post analyses were not performed. The model was tested in an infant neuropsychiatry unit, but extension of the model to other units should be achieved in the future.

Further studies could also try to extend the model to other healthcare contexts to test its effectiveness. Moreover, different models could be implemented through the DES approach to handle absenteeism to evaluate the most efficient and effective model since overbooking is not the only strategy available. Nevertheless, the combination of agile and Six Sigma proved to be useful in and feasible for analysing simulation models in healthcare.

## Figures and Tables

**Table 1:** Parameters of the Infant Neuropsychiatry Unit.

Medium Wait (Days)	Max Wait (Days)	Min Wait (Days)	Chaos (%)	Inefficiency (%)
208	421	0	56	85

**Table 2:** One-way ANOVA results. (DF = Degree of Freedom; Adj SS = Adjusted sums of squares; Adj MS = Adjusted mean squares)

**Bookings Per Month**					
	**DF**	**Adj SS**	**Adj MS**	**F-Value**	***p*-Value**
Month	11	837.2	76.106	7.86	0.000
Error	256	2477.4	9.677		
Total	267	3314.6			
**Bookings Per Day**					
	**DF**	**Adj SS**	**Adj MS**	**F-Value**	***p*-Value**
Day	6	62.64	12.527	2.00	0.079
Error	261	1632.02	6.253		
Total	267	1694.66			

**Table 3:** One-way ANOVA model summary.

	S	R^2^	R^2^ (adj)	R^2^ (pred)
Bookings per month	3.11084	25.26%	22.05%	18.23%
Bookings per day	2.50059	3.70%	16.5%	14.6%

**Table 4:** The level of overbooking increases, the time to work through the queue is reduced, and as a result, the reference time sample is reduced for the calculation of the number of visits above (#Over) or below (#Under) the expected availability.

Scenario	Overbooking Levels	#Under	#Over	Average Under	Average Over	Diff.	Equilibrium	Steady Time
1	18	118	35	−3.52	2.63	0.89	−2.12	615
2	19	107	39	−3.34	2.71	0.63	−1.73	581
3	20	62	34	−3.27	3.02	0.25	−1.13	551
4	21	74	47	−3.16	3.06	0.10	−0.74	523
5	22	60	48	−3.15	3.48	0.33	−0.20	495
6	23	52	51	−2.94	3.67	0.73	0.33	474
7	24	44	53	−2.77	3.74	0.97	0.78	454
8	25	32	52	−2.88	4.10	1.22	1.44	432

**Table 5:** Scenarios 4–7 are the only scenarios that take into consideration the presence of the value of equilibrium.

	Scenario 4	Scenario 5	Scenario 6	Scenario 7
Tot. occurrences	43	48	51	54
Tot. useful days	133	123	112	104
% occur./useful days	32%	39%	46%	52%

**Table 6:** Adding overtime parameters to scenario 4–7 as “covered visits”.

	Scenario 4	Scenario 5	Scenario 6	Scenario 7
Covered visits	27	19	20	19
Tot. occurrences	18	29	31	35
Tot. useful days	139	123	112	104
% occur./useful days	13%	24%	28%	34%
